# Combined nerve and tendon transfer strategy for the restoration of grasp in tetraplegia; a case report

**DOI:** 10.1038/s41394-024-00695-6

**Published:** 2025-01-06

**Authors:** Lina Bunketorp Käll, Johanna Wangdell, Carina Reinholdt, Jan Fridén

**Affiliations:** 1 Center for Advanced Reconstruction of Extremities (CARE), Sahlgrenska University Hospital/Mölndal, Mölndal, Sweden; 2 Swiss Paraplegic Centre, Nottwil, Switzerland

**Keywords:** Outcomes research, Quality of life, Motor neuron, Neurophysiology, Spinal cord diseases

## Abstract

**Introduction:**

By combining nerve and tendon transfer procedures, a more versatile hand function can be expected. Here we report the long-term outcomes of novel, individualized reconstruction strategies using combined nerve and tendon transfer procedures (CNaTT) to restore prehension and grasp in two patients with tetraplegia.

**Case presentation:**

Two women, 45 years of age, underwent bilateral nerve transfer according to the Bertelli S-PIN (supinator to posterior interosseous nerve transfer) procedure. The grip reconstruction included tendon transfers using brachioradialis to flexor pollicis longus and extensor carpi radialis longus to flexor digitorum profundus, as well as balancing tenodesis, arthrodesis procedures and intrinsic reconstruction. At 6 months, the patients’ pinch and grasp strength ranged between 1.0–2.0 and 2.2–5.0 kg, respectively, concomitant with improvements in activity and occupational performance. At 4–7 years after the grip reconstruction, both patients had full metacarpophalangeal (MCP) extension scoring M5 and M4, as well as full thumb extension scoring M5 and M4 on the right side. On the left side, MCP extension was weaker for both patients (M1/M2), whereas the thumb could extend against gravity (M3/M4). The maximal 1^st^ webspace opening measured between 5 and 11 cm. Pinch strength measured between 1.25 and 2.6 kg, and whole hand grip strength between 3.9 and 7.8 kg. The patients’ grasps could fit around 80 and 50 mm wide cylinders using a normal right-handed grasp.

**Discussion:**

The CNaTT procedure successfully restored useful grasp and release function with long-lasting effects. A large-scale controlled study is needed to confirm these findings.

## Introduction

Pioneering work in the 70 s guided the development of reconstructive upper limb surgery in tetraplegia [1, 2]. Traditionally, the reconstruction strategy involves tendon transfers [3]. This procedure allows reliable restoration of finger and thumb flexion and increases the degree of personal freedom, e.g., the ability to grasp, pick up and manipulate objects [2, 4]. Normally, the tendon transfer, however, requires a passive opening of the hand to grasp objects. Many daily life activities, however, involve active opening of the hand.

Nerve transfer techniques expand the toolbox of reconstructive surgeries available for persons with tetraplegia [3, 5–11]. By combining nerve and tendon transfer procedures, a more versatile hand function can be expected. Although the utility of nerve transfers has yet to be fully determined, several studies have demonstrated successful reinnervation of paralyzed target muscles [9, 12]. It has been suggested that research should be directed at evaluating combined tendon and specific nerve transfer techniques, such as the Bertelli S-PIN (supinator to posterior interosseous nerve transfer) procedure [13]. The S-PIN procedure can reanimate not only the finger extensors but also allows independent thumb extension and abduction [9] as well as stabilizing and balancing the wrist, thereby enabling the active opening of the hand [13]. The S-PIN procedure combined with the tendon transfer technique has the unique potential to restore both active opening and closing of the hand in patients with tetraplegia. This strategy, hereafter referred to as Combined Nerve and Tendon Transfer (CNaTT), potentially maximizes the functional gains more than either technique in isolation. In this case report, we present the outcome of the first CNaTT procedures performed to restore prehension and grasp and improve hand function in two patients with tetraplegia.

## Case presentation

**Fig. 1:**
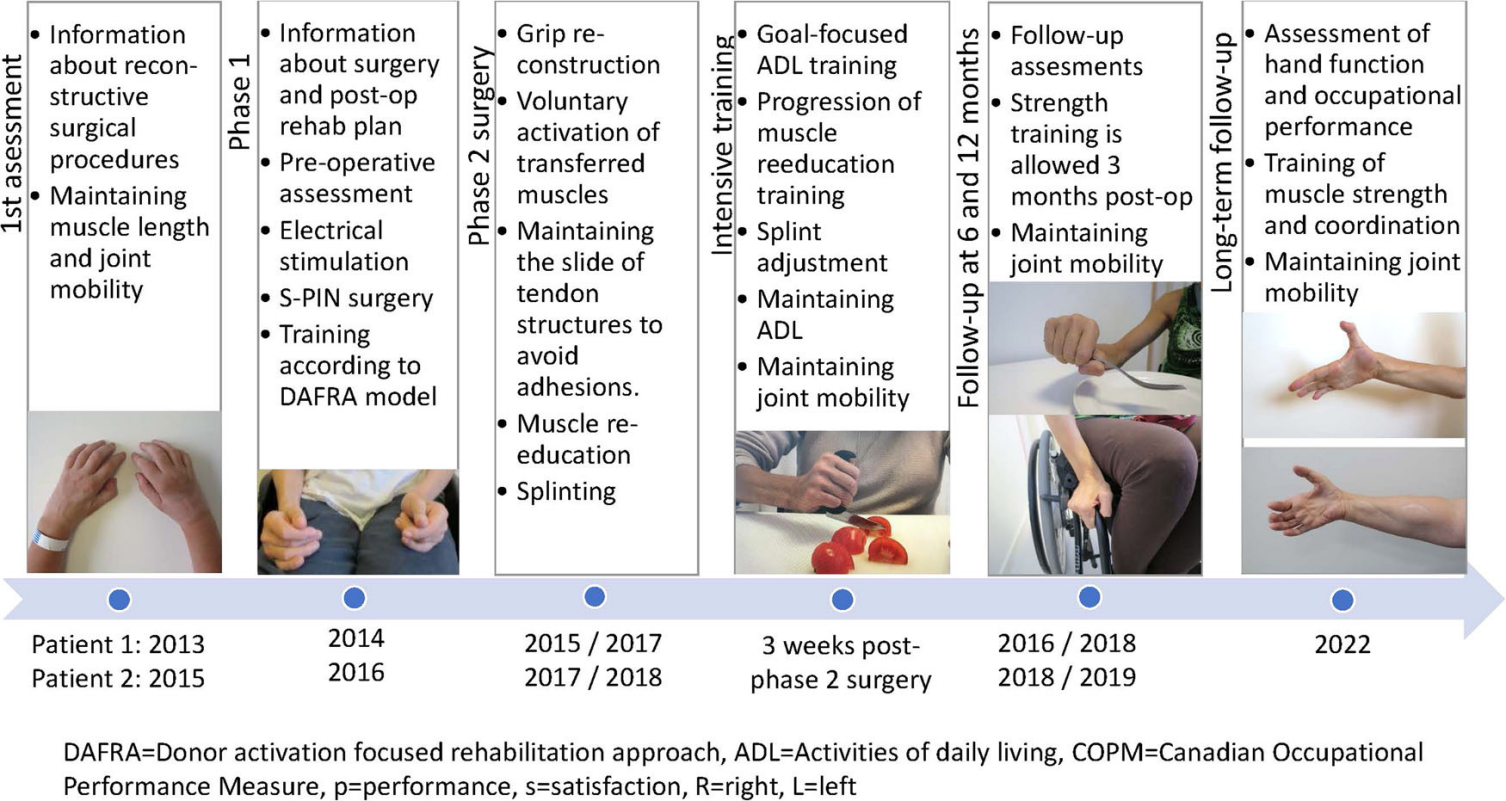
Timeline of the combined nerve and tendon transfer procedure from the 1^st^ assessment to the long- term follow-up.

This case study presents the details of the surgical procedures completed for two patients with tetraplegia. The study was approved by the Swedish Ethical Review Authority (reference number: 2019-05838), and the participants gave written informed consent. A timeline of the CNaTT procedure and follow-ups is presented in Fig. 1.

### Patient 1

Patient 1 was a 45-year-old woman who sustained a C5-C6 American Spinal Injury Association (ASIA) grade A [14] cervical spinal cord injury (C-SCI) leading to complete tetraplegia following a horseback riding accident in November 2013. After anterior fusion and rehabilitation, she was left with complete paralysis distal to the wrists and more innervated muscles below the elbow in the right as compared to the left arm (Table 1).

**Table 1:** Clinical characteristics of patients prior to the combined nerve and tendon transfer procedure.

Variable	Patient 1 *Right*	Patient 1 *Left*	Patient 2 *Right*	Patient 2 *Left*
ICSHT	5	3	4	4
*Muscle strength (MRC 0–5)*				
Triceps	3	1	5	5
BR	5	3	5	5
ECRB/ECRL	5	4	5	5
PT	5	0	4	3
FCR	3	1	0	0
APL, EPL, EDC and ECU	0	0	0	0

*BR* brachioradialis, *MRC* Medical research council, *ECRL* extensor carpi radialis longus, *ECRL* extensor carpi radialis brevis, *PT* pronator teres, *FCR* flexor carpi radialis, *APL* abductor pollicis longus, *EPL* extensor pollicis longus, *EDC* extensor digitorum communis, *ECU* extensor carpi ulnaris, *ICSHT* International Classification of Hand Function in Tetraplegia.

#### Surgery - phase 1 (nerve transfer)

Eleven months after the injury, the patient underwent bilateral S-PIN transfer [13]. Prior to surgery, neurophysiological assessments verified that both the donor and recipient muscles were excitable on both sides, although higher stimulation amplitude was required on the left side compared to the right (1.5 vs. 0.5 mA).

#### Follow-up post-S-PIN

At 7 months post-surgery, patient 1 demonstrated the first signs of reinnervation on the right side, i.e., a weak but clearly detectable extension of the thumb and extension of the MCP joints of all fingers. She also reported better stability of the wrist with less radial deviation. At 9 months, the extension and radial abduction of the right thumb were graded as M3, and the extension of the right MCP and PIP joints were graded as M3, including the independent extension of MCP V (EDM). A distinct activation of the ECU was detected at this point. On the left side, the first signs of reinnervation were observed, with an active extension of the thumb and the two radial fingers together with a less pronounced radial deviation of the wrist when extended.

#### Surgery - phase 2 (tendon transfers and tenodesis)

Twelve months after the nerve transfer patient 1 underwent reconstruction of grip functions on the right hand. The surgery included tendon transfers using BR to FPL, ECRL to FDP (2–4), and, notably, the reinnervated EDM to APB. The objective of these procedures was to provide active key pinch, finger flexion, and palmar abduction of the thumb. In order to prevent claw handing but without jeopardizing the integrity of the newly reinnervated finger extensors, tenodeses at the MCP level, according to Zancolli-lasso, were performed for all four fingers [15]. Thirty months after the nerve transfer, patient 1 had surgery on the left hand with the aim of restoring passive pinch grip and active finger flexion. The surgery included tendon transfer of BR to FDP, passive tenodeses at the MCP level according to Zancolli-lasso on fingers 2, 3, and 4, arthrodesis CMC-1, tenodesis of FPL to the distal radius, and shortening of the distal ECU tendon. A summary of the pre-and post-operative rehabilitation regimen is presented in Supplement 1.

### Patient 2

Patient 2 was a 45-year-old woman who sustained complete tetraplegia due to a C6 ASIA grade A [14] C-SCI following a bike accident in October 2015. After rehabilitation, the patient was left with complete paralysis distal to the wrists, with nearly identical residual motor function in the right and left arms (Table 1).

#### Surgery - phase 1 (nerve transfer)

Ten months after the injury, the patient underwent the bilateral S-PIN procedure [13]. By means of an intra-operative nerve stimulator, the supinator donor nerve was shown to be excitable at 0.5 mA on both sides. On the left side, the extensor carpi ulnaris (ECU) was excitable, whereas thumb and finger extensors were not. On the right side, the ECU and thumb extensor nerves were excitable, but the finger extensors were not.

#### Follow-up post-S-PIN

At 6.5 months post-surgery, patient 1 demonstrated clear signs of reinnervation on the right side, including a detectable extension of the MCP joints of all fingers and traces of reinnervation in the thumb and stabilized ECU tendon, with less tendency of radial deviation in the wrist when extended. On the left side, a weak but detectable thumb reinnervation was found. At 9 months, the strength in the right-side MCP joints and thumb extension were graded M3. A somewhat more distinct activation of the ECU was detected at this point. On the left side, the first signs of reinnervation with an active MCP joint and thumb extension were observed, together with a less pronounced radial deviation of the wrist when extended.

#### Surgery - phase 2 (tendon transfers, tenodesis, and arthrodesis)

Thirteen months after the nerve transfer and with M2–3 strengths for all reinnervated muscles, patient 2 underwent reconstruction of the grip functions of the right hand. To provide active key pinch and finger flexion, tendon transfers were performed using BR to FPL and ECRL to FDP (2-4). Split FPL– extensor pollicis longus (EPL) distal thumb tenodesis was done to prevent hyperflexion of the interphalangeal (IP) joint of the thumb [16], along with EPL to EPB tenodesis. To optimize the direction of thumb action against the index finger, capsulodesis of the first carpometacarpal (CMC) joint was done. To optimize hand opening, the interossei function for all fingers was reconstructed according to McCarthy et al. (House procedure) [17] to achieve passive extension in the PIP joints. Phase 2 surgery was repeated for the left arm, including identical tendon transfer procedures and tenodeses as for the right arm.

## Results

### Follow-up at 6 and 12 months

**Fig. 2:**
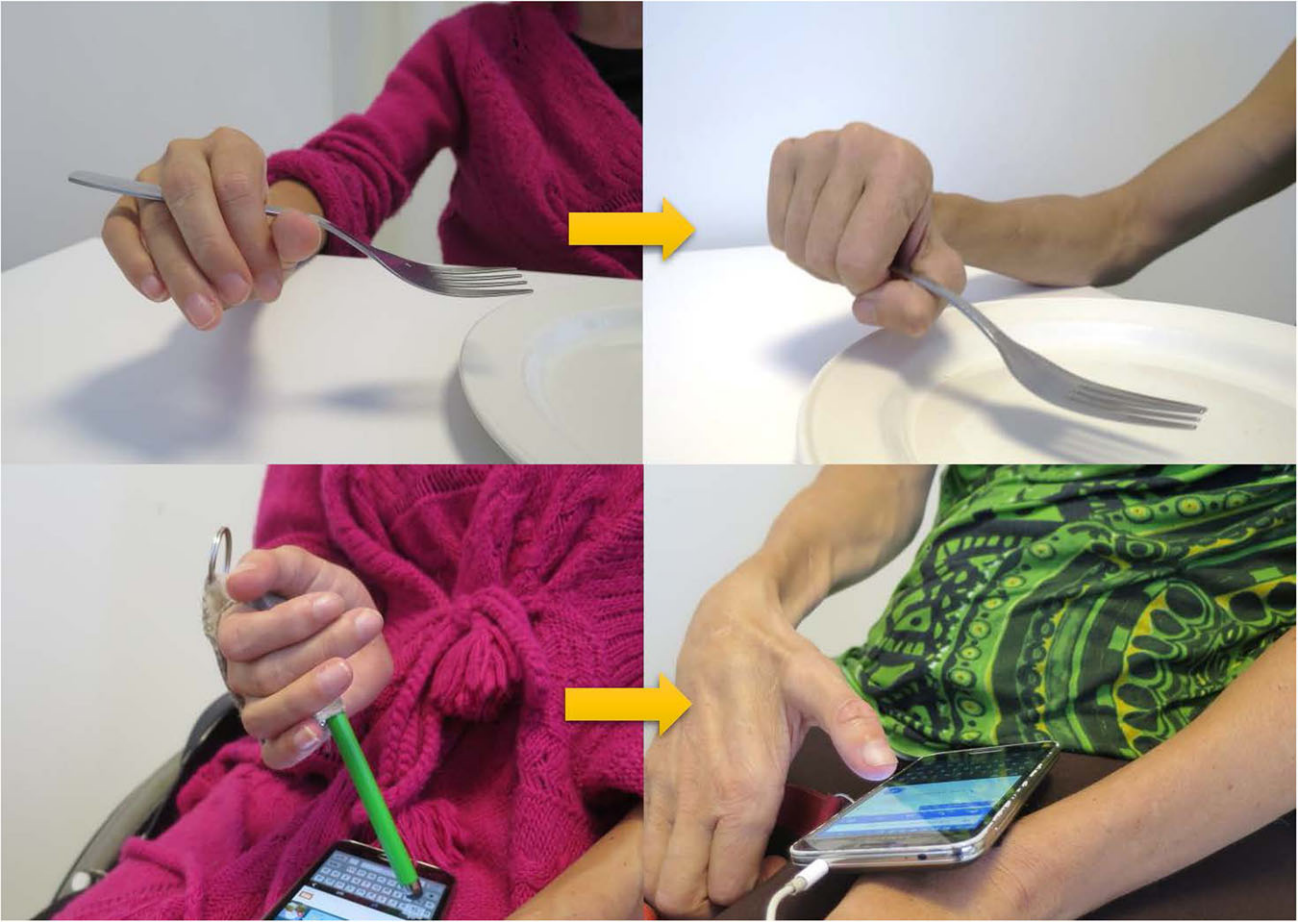
Hand appearance before and 6 months after phase 2 grip reconstruction for patient 1.

**Fig. 3:**
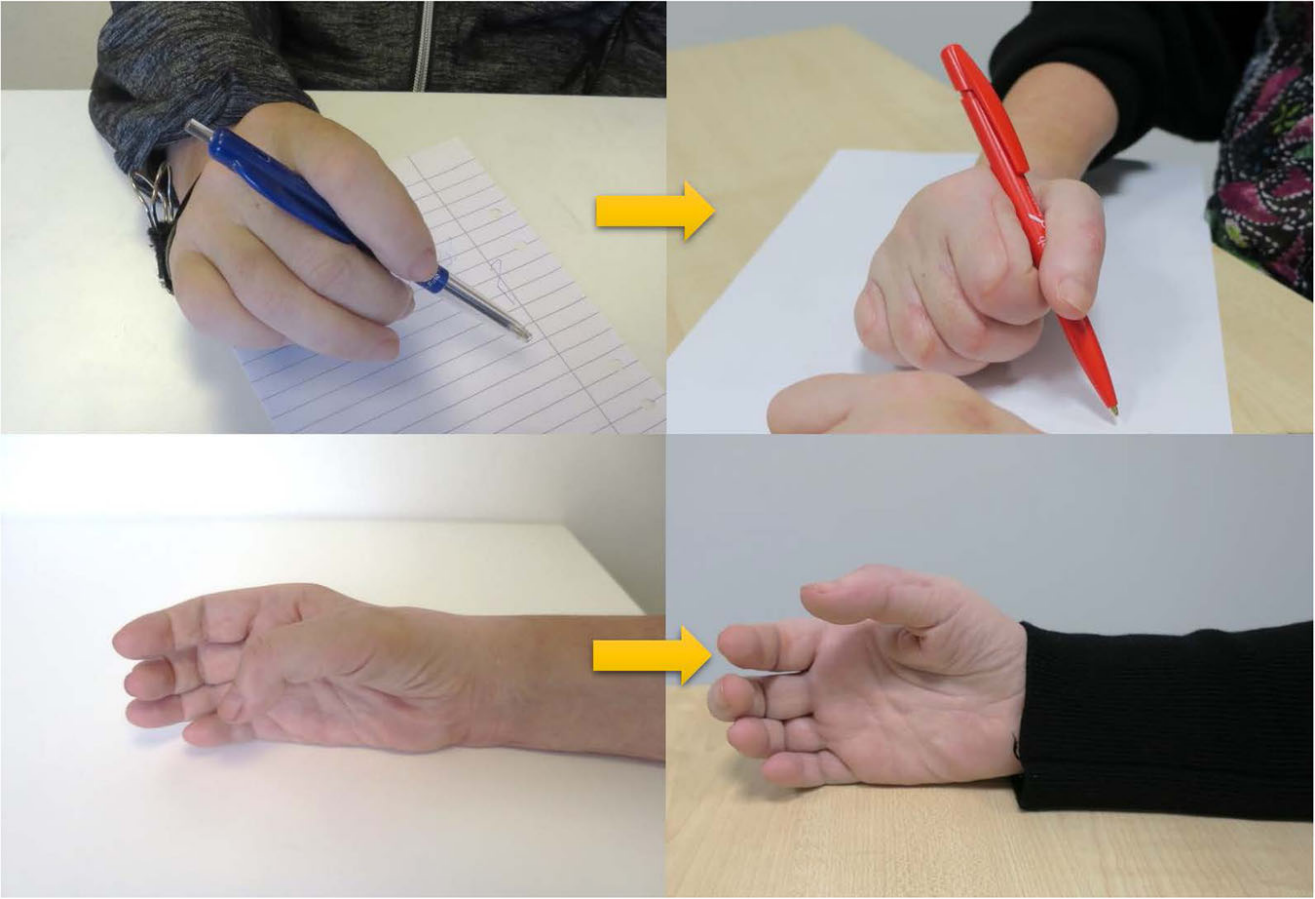
Hand appearance before and 6 months after phase 2 grip reconstruction for patient 2.

For patient 1, grip strength, grasp, activity and occupational performance had increased 6- and 12- months after phase 2 surgery (Table 2). Activations of both the reinnervated and tendon transfer-powered motor, especially in the right hand, were prompt (Supplementary File 2 and Fig. 2). At the 12-month follow- up, patient 1 had returned to work as a veterinarian on a part-time (50%) basis. For patient 2, grip strength, grasp, and activity performance had increased, concomitant with improvements in occupational performance (Table 3, Fig. 3). The prompt finger and thumb extension allowed a web space opening large enough to grasp a bottle, and restored grip functions made it possible to pour from the bottle into a glass (Supplementary File 3). In 2019, patient 2 initiated vocational rehabilitation, and starting mid-year 2021, she returned to work in a sales business on a half-time basis.

**Table 2:** Follow-up data for patient 1 at 6- and 12-months after the phase 2 grip reconstruction.

Variable	RIGHT 6 months	RIGHT 12 months	LEFT 6 months	LEFT 12 months
PINCH max (kg)	2.0 (0)	1.8 (0)	1.0 (0)	0.7 (0)
JAMAR max (kg)	5.0 (0)	4.0 (0)	3.5 (0)	3.7 (0)
Rating-hand function (VAS)	6 (2.8)	10 (2.8)	2.2 (1)	3 (1)
GRT total	126 (70)	169 (70)	70 (13)	70 (13)
COPM performance^a^	3.6 (1.2)	3.6 (1.2)	5.6 (1.6)	5.6 (1.6)
COPM satisfaction^a^	4.0 (1.0)	4.0 (1.0)	5.5 (2.4)	5.5 (2.4)

Data within brackets are from the assessment before the phase 2 surgical procedure.

*VAS* Visual Analogue Scale (0–10), *GRT* Grasp and Release Test: The GRT is a pick-and-place test that requires the participant to unilaterally acquire, move, and release 6 objects varying in size and weight. The total number of repetitions achieved in a 30-second trial for all 6 objects were recorded.

^a^*COPM* Canadian Occupational Performance Measure: Patients were asked to report up to 5 of the most relevant activity limitations. They were also asked to rate each performance level in the prioritized activities prior to surgery on a scale ranging from 1 to 10. Patients were then requested to rate their satisfaction with their performance in the prioritized activities using the COPM, Satisfaction aspect.

**Table 3:** Follow-up data for patient 2 at 6- and 12-months after the phase 2 grip reconstruction.

Variable	RIGHT 6 months	RIGHT 12 months	LEFT 6 months	LEFT 12 months
PINCH max (kg)	1.0 (0)	1.0 (0)	1.0 (0)	1.0 (0)
JAMAR max (kg)	2.2 (0)	4.0 (0)	5.0 (0)	7.0 (0)
Rating-hand function (VAS)	3 (1)	5 (1)	5 (1)	5 (1)
GRT total	140 (73)	167 (73)	91 (28)	127 (28)
COPM performance	4.6 (1.4)	6.0 (1.4)	4.0 (1.4)	4.2 (1.4)
COPM satisfaction	5.4 (1.4)	5.8 (1.4)	3.6 (1.4)	4.2 (1.4)

Data within brackets are from the assessment before the phase 2 surgical procedure.

*VAS* Visual Analogue Scale, *GRT* Grasp and Release Test, *COPM* Canadian Occupational Performance Measure.

### Long-term follow-up

**Fig. 4:**
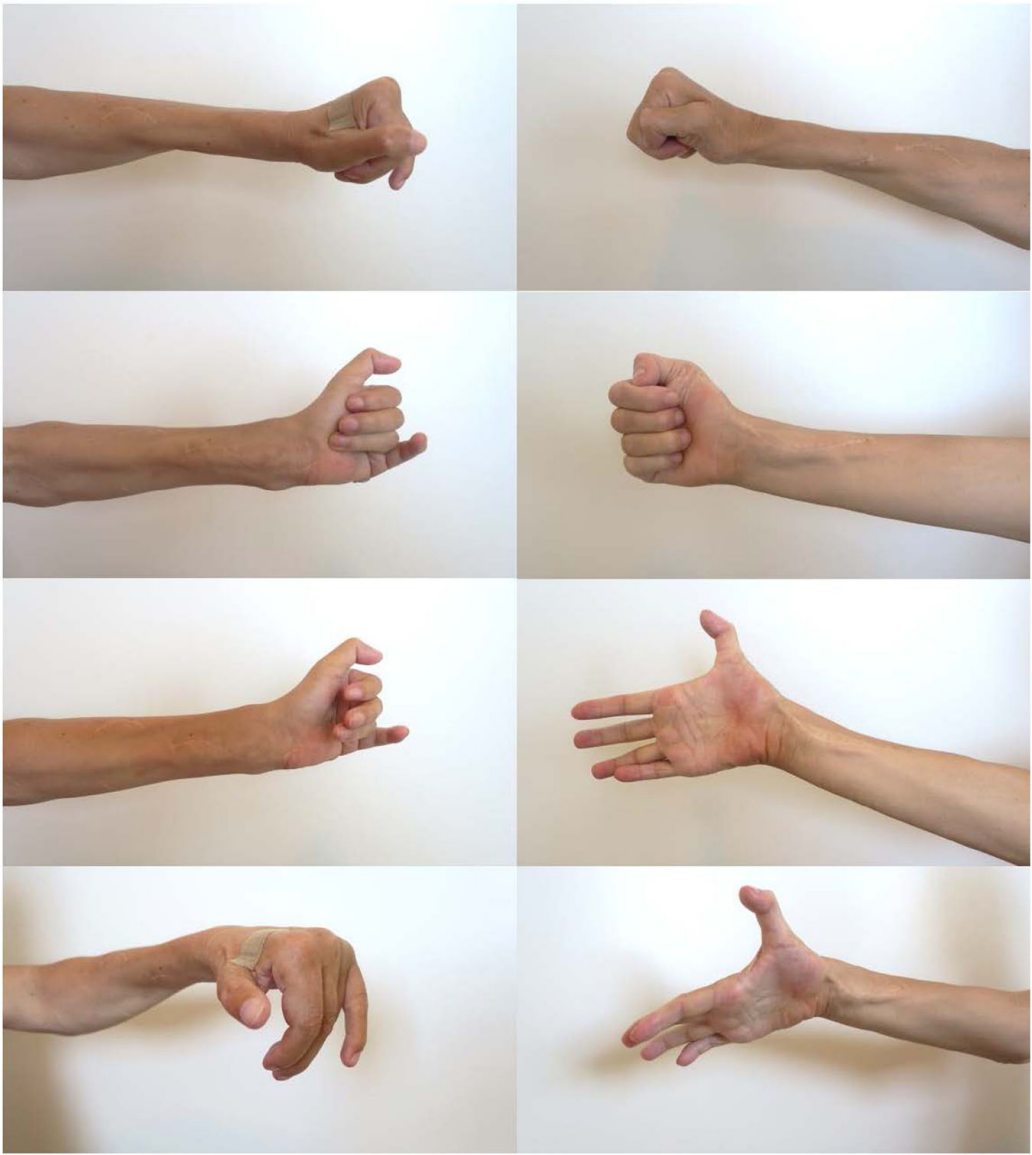
Photographs taken of the long-term functional results of the combined nerve and tendon transfer procedures enabling varying degrees of whole hand and first webspace opening for patient 1.

**Fig. 5:**
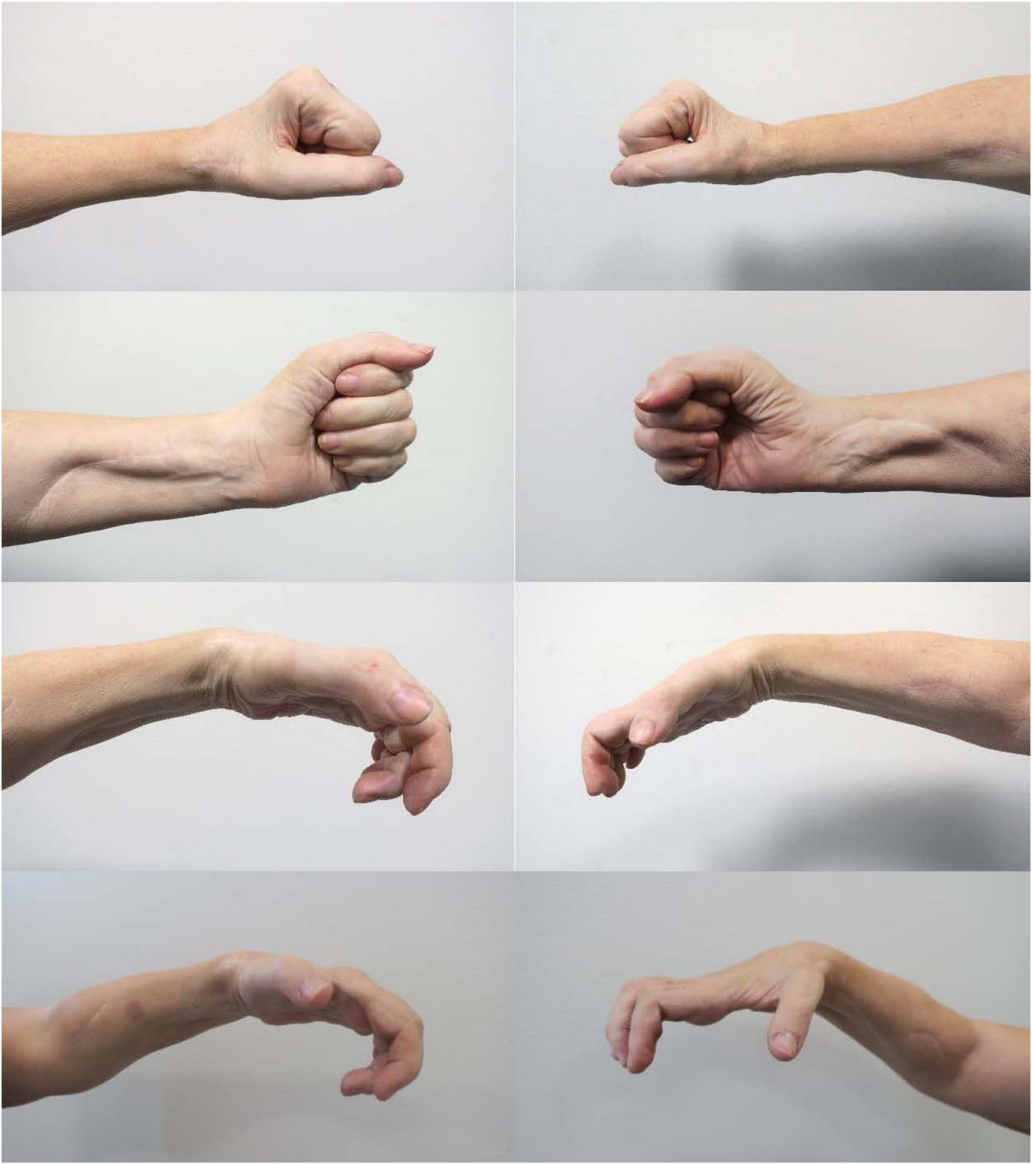
Photographs taken of the long-term functional results of the combined nerve and tendon transfer procedures enabling varying degrees of whole hand and first webspace opening for patient 2.

For patient 1, the long-term follow- ups took place 7 and 5 years post-phase 2 surgery of the right and left hands, respectively (Fig. 1). On the right side, the strength in the reinnervated muscles allowed a prompt opening of the hand and webspace (Fig. 4). On the left side, the thumb could extend against gravity (M3), and the finger extensors had only weak signs of reinnervation (M1), with a resulting limited hand opening (Fig. 4). The thumb pulp could be separated 11 and 5 centimeters from the index pulp on the right and left hand, respectively (Table 4). The gains in grip strength, grasp, activity and occupational performance was sustained and the patient was capable of grasping an 80 mm wide cylinder object with the right hand as measured by the cylinder test whereas only a weak and limited hand opening was achieved with the left hand (Table 4). For patient 2, the long-term follow-up took place 5 and 4 years post-phase 2 surgery of the right and left hands, respectively (Fig. 1). The strength in the reinnervated finger and thumb extensors in the right hand had reached grade M4, and the thumb abductor grade was M3, allowing an opening of the hand and first webspace (Fig. 5). The finger extensors of the left hand were weaker (M2), and the thumb abductor showed no signs of reinnervation, whereas the thumb extensor had reached grade M4 with a resulting functional hand opening (Table 4). The thumb pulp could be separated 8 and 5 centimeters from the index pulp on the right and left sides, respectively. The grip strength, grasp, activity and occupational performance achieved at the long-term follow-up are presented in Table 4. With the right hand patient 2 could grasp twice as wide cylinder objects as the left hand (50 vs. 20 mm for subtest 1).

**Table 4:** Follow-up data for patients 1 and 2 at 4–7 years after the phase 2 grip reconstruction.

Variable	Patient 1 RIGHT *7* *yrs post-grip rec*	Patient 1 LEFT *5* *yrs post-grip rec*	Patient 2 RIGHT *5* *yrs post-grip rec*	Patient 2 LEFT *4* *yrs post-grip rec*
Muscle strength				
ECRL/B (0–5)	5 (5)	4 (4)	5 (5)	5 (5)
ECU (0–5)	5 (2)	0 (0)	2 (1)	1 (0)
EDC (0–5)	5 (3)	1 (1)	4 (3)	2 (1)
EPL (0–5)	5 (3)	3 (3)	4 (2)	4 (1)
APL (0–5)	1 (2)	0 (0)	3 (0)	0 (0)
PINCH max (kg)	1.50 (0)	1.25 (0)	2.25 (0)	2.60 (0)
JAMAR max (kg)	5.5 (0)	3.9 (0)	7.8 (0)	7.2 (0)
Opening thumb to index fingertip (cm)	11.0 (10)	5.0 (1)	8.0 (7)	5.0 (5)
Opening thumb to index PIP-joint (cm)	8.0 (6)	4.5 (1)	7.0 (7)	5.0 (3.5)
Radial deviation – resting position (°)	0 (10)	15 (0)	15 (10)	20 (15)
Radial deviation –while extending the wrist (°)	0 (20)	20 (30)	20 (25)	30 (20)
Flex IP-joint at rest (°)	90 (0)	55 (0)	30 (40)	40 (45)
Flex IP-joint at pinch (°)	95 (90)	90 (0)	30 (45)	30 (80)
Rating-hand function (VAS)	7 (3)	4 (1)	8 (1)	6 (1)
Cylinder Test^a^				
one-hand normal (mm)	80	0	50	20
one-hand adapted (mm)	90	20	70	30
two-hand (mm)	90	30	80	70
placing (mm)	130	70	120	80
GRT total	186 (70)	76 (13)	168 (73)	156 (28)
COPM performance	6.80 (1.2)	6.75 (1.6)	9.00 (1.0)	5.40 (1.4)
COPM satisfaction	6.80 (1.0)	7.50 (2.4)	9.60 (1.0)	6.00 (1.4)

Data within brackets are from the assessment before the phase 2 surgical procedure.

*Yrs* years, *ECRL* Extensor Carpi Radialis Longus, *ECRB* Extensor Carpi Radialis Brevis, *ECU* Extensor Carpi Ulnaris, *EDC* Extensor Digitorum Communis, *EPL* Extensor Pollicis Longus, *APL* Abductor Pollicis Longus, *PIP* proximal interphalangeal, *IP* interphalangeal, *VAS* Visual Analogue Scale, *GRT* Grasp and Release Test, *COPM* Canadian Occupational Performance Measure.

^a^Cylinder Test: The test comprises four subtests that measures the capacity to perform an active or passive cylinder grip in either a normal, adapted, self-assisted or examiner-assisted way (placing). Each subtest is scored 20 to 150, with larger scores representing better/larger hand opening.

## Discussion

These two cases demonstrate excellent long-term functional results when combining nerve and tendon transfer procedures to restore grip functions in individuals with tetraplegia. Overall, the functional gains demonstrated one year after the phase 2 procedure did persist and were, to some extent, even improved at the long-term follow-up. The active thumb and finger extensor function obtained in this reconstruction are usually only observed in lower cervical lesions, i.e., C7-C8 levels. In other words, the outcome of the S-PIN transfer may be interpreted as a recovery from ICSHT 5 to ICSHT 8 according to the international classification. Special considerations must be taken for the intrinsic balancing of the fingers. In a previous kinematic study, it was demonstrated that both intrinsic balancing techniques improve grasp, but only the House procedure restored a hand kinematic approximating those of an intrinsic activated hand, which is why this technique is advocated for individuals with tetraplegia [18]. However, with successful reinnervation of extrinsic finger extensors, we believe it contradictory to interfere with the extensor apparatus of the fingers. On the other hand, successful reinnervation of the EDM allows for transfer to the APB to enable palmar abduction and pronation of the thumb. This reconstruction requires limited strength and represents primarily a postural function. Patient 1 demonstrated sufficient active MCP and concomitant PIP extension with the wrist in neutral, i.e., without any passive tenodesis effect extending the fingers. Based on this observation, a Zancolli-lasso tenodesis [15] was undertaken for all four finger MCP joints to prevent claw handing without surgically intervening with the finger extensor mechanism. For patient 2, PIP extension was not sufficient, which is why intrinsic function for all fingers was reconstructed by a House procedure [17] to optimize hand opening by passive extension in PIP joints.

However, given the limitations that both tendon and nerve transfer procedures present, it is critical to find the optimal timing of the two procedures, whether done alone or, more importantly, in combination. The optimal timing for the two procedures is debatable. For phase 1, we adhered to the recommendations presented in previous case reports and case series, which indicated better functional outcomes when nerve transfers are performed within a year after the C-SCI [2]. However, at the recent symposium “Nerve Transfers in the Upper Limb” organized by the Institute de la main in Paris May 2023, the expert opinion was to perform the S-PIN transfer as early as possible even when the excitability of target muscles was weak or absent. Providing the supinator is under full voluntary control, the short nerve coaptation to muscle target distance for the S-PIN implies a reasonable probability of reinnervation. The nearest target is the ECU. A reinnervation of this muscle corrects the radial deviation of the wrist and establishes a straighter and biomechanically more favorable line of action for the main wrist extensor (ECRB, extensor carpi radialis brevis).

The timing of phase 2 is not as critical as that of phase 1. The patient should, however, be informed about the necessity of undergoing a second surgery due to the change of finger extensor- flexor balance after successful reinnervation of the PIN-innervated muscles. The posture of the hand has changed, and the increased tone of the thumb and finger extensors will impair whatever tenodesis hand grip achieved during the early rehabilitation.

In a previous case report [19], Bertelli et al. presented the results of a patient who underwent nerve and tendon transfers in a single surgery 18 months after a complete C-SCI. In addition to the S-PIN procedure, the BR tendon was transferred to the FPL and flexor superficialis of the index finger. Twenty-two months after surgery, the patient had full MCP joint extension (M4) in both hands and pinch strength of 2.0 and 1.5 kg on the right and left hand, respectively. The strength in the right and left thumb extensors was graded M3 and M4, respectively. The thumb pulp could be separated 53 and 66 mm from the index distal phalanx on the right and left sides, respectively.

It appears highly reasonable to await the outcome of nerve transfers and, thereafter, depending on the extent of reinnervation, tailor tendon transfer procedures to maximize functional outcomes. This approach certainly has its drawbacks because of the time delay until full, partial, or absent reinnervation can be evaluated. Successful nerve transfers provide the patient with a more ‘normal’ motor control of the restored extrinsic muscle functions than that attained by tendon transfers. However, in light of the recent study by Javeed et al. [20] demonstrating that 52% of hands gain an MRC grade of 3 or higher for finger flexion, a tendon transfer backup may be considered for the remaining 48%. In summary, this case study reports a logical and reliable strategy of combining a relatively simple nerve transfer of predictable outcome with modified but science- and experience- based tendon transfers and tenodesis approaches.

## Conclusion

In this case report, a CNaTT procedure successfully restored useful grasp and release function and increased occupational performance. The patients continued to demonstrate functional gains at the long-term follow-up concomitant with increased performance of daily activities, suggesting long- lasting effects. Our findings suggest that adding a nerve transfer to the traditional tendon transfer procedure is a safe and technically feasible means for upper limb reanimation. Although these results are encouraging, a large-scale controlled study is needed to confirm these findings and, if confirmed, determine the optimal timing of the two procedures.

## Supplementary information

Supplemental Material 1

Supplemental Material 2

Supplemental Material 3
