# Supplementary material for: Combined nerve and tendon transfer strategy for the restoration of grasp in tetraplegia; a case report
Source: Spinal Cord Ser Cases. 2025 Jan 6;11:1. doi: 10.1038/s41394-024-00695-6 (PMC11704322; doi:10.1038/s41394-024-00695-6)
Supplement: Supplementary file 1 — Supplemental Material 1 [file 41394_2024_695_MOESM1_ESM.docx]

**SUPPLEMENT 1**

**Summary of the pre-and post-operative rehabilitation regimen**

*Pre-surgical assessment*

Patients who meet the criteria for nerve transfer regardless of the time that has passed since injury undergo electric stimulation assessment to distinguish between paralyzed muscles with intact versus damaged lower motor neurons (LMNs). In the case of LMN damage, the muscle fibres receive week or no nerve impulses, causing the motor end plates of the muscle to disappear. All recipient muscles are stimulated according to an established mapping system.^1^ Its ability to contract is graded as normal, impaired or absent. When stimulating the motor points of muscles affected by LMN damage they respond weakly or not at all.^2^ It is then particularly important that the nerve transfer is done within one year after the injury since prolonged denervation causes degeneration of myofibrils and, eventually, death of the muscle cells and motor end plates.^3^ In the case of an upper motor neuron injury, a preserved reflex arc is likely to excite the motor end plates, so that a nerve transfer can restore function even at a later stage. In some cases, the spinal cord injury may include a combination of upper and lower motor neuron damage.

*Pre-surgical information and exercise instructions*

Providing patients with adequate information and education is important in the preparatory phase. It needs to include the relevance of maintaining muscle length and joint mobility, as well as the importance of engaging the upper limbs in daily activities to maintain residual motor functions. The patient also needs to be made aware that the combined nerve and tendon transfer (CNaTT) procedure is a two-stage procedure, in which the first nerve transfer phase aims at reanimating hand opening and does not improve grip ability, but rather risks deteriorating any well-functioning tenodesis grip. It is not until after phase two (grip reconstruction) that activity gains can be expected. Before undergoing a nerve transfer, the patient is taught exercises to condition the nerve supplying the donor muscle. The purpose is to promote activity in the sensory-motor cortex area and to boost the interconnectivity and neural pathway from the motor cortical network to the specific nerve that will grow into the peripheral recipient muscles. In the case of volitional control in pronator and wrist flexors, those muscles should be strengthened as they serve as important antagonist to finger and thumb extensors once those get reanimated. Any issues of muscle length, joint mobility and edema are addressed with splinting, positioning, and exercise, in order to optimize the conditions for the reconstructive procedures.

*Post-operative care and training regimen after phase 1 surgery (S-PIN)*

After a S-PIN procedure, tensioning the transferred nerve is not allowed why certain restrictions after surgery could be applied such as limiting full elbow extension and supination. During the first two weeks, the arms should not be fully loaded. To protect the surgical area and avoid tensioning of the nerve, patient may need to wear a splint the first two weeks. The training regimen is inspired by the Donor activation focused rehabilitation approach (DAFRA).^4^ The DAFRA model recognizes the altered neural pathways created by the nerve transfer and focuses on maximizing functional outcomes by strengthening these neural pathways.

*Post-operative care and training regimen after phase 2 surgery (grip reconstruction by tendon transfer techniques)*

After wound cleaning and change of dressing, patients are equipped with a standard splint with the wrist in approximately 30° of extension, fingers in approximately 60° of MCP flexion and extended PIP and DIP joints. The splint is worn 24 hours a day for three weeks, but is removed for training sessions 4 times per day, under careful supervision starting on the first post-operative day.^5^ The advanced suture technique allows the transferred tendons to be exercised immediately after surgery. Voluntary activation of a transferred tendon is facilitated by instructing the patient to recruit the function of the donor muscle. For example, when brachioradialis (BR) is transferred to replace the action of the flexor pollicis longus (FPL) muscle, the patient should perform flexion of the elbow against resistance, with the forearm in a semi-pronated position and the thumb passively extended. External resistance has empirically been shown to increase muscle activation and makes it easier to achieve the full active ROM allowed.^6^ The isometric recruitment of the BR muscle then acts as the new thumb flexor achieving the restored pinch grip. Multiple daily training sessions are important to prevent adhesions, but also to facilitate successful recruitment of restored function and, supposedly, to promote cortical remapping. In the motor re-learning process, visual perception and sensation are thought to be of importance why patients are instructed to focus their sight on the specific function that is trained, i.e. pinch- and whole hand grasp. As soon as the patient is capable to activate the pinch grip by just thinking of the target movement, which requires co-contraction of the triceps muscle, they should no longer voluntarily focus on the function of the donor muscle. Thus, external resistive force is no longer necessary, which implies that the brain has adapted to the peripheral redirection of tendons. If a patient has trouble in recruiting the reconstructed function the previous training strategy is resumed, in which focus is put on the function of the donor muscle, which is recruited against resistance. After discharge, patients must fully comply with the restrictions and training regimen and perform the training program by themselves or with help from an assistant. Patients are discharged on the fourth day after surgery. Three weeks later they return to the clinic for a week of in-house training. At this stage, the aim is to progress training of grip functions and to start using the restored functions in activities of daily living. From now on, the splint is worn only during the night, for a total of three months since the surgery. Not until three months after the phase 2 grip reconstruction, patients are allowed to fully load the transferred tendons.

**References**

1. Bersch I, Koch-Borner S, Fridén J. Electrical stimulation—a mapping system for hand dysfunction in tetraplegia. *Spinal Cord*. 2018;56(5):516-522.

2. Bryden AM, Hoyen HA, Keith MW, Mejia M, Kilgore KL, Nemunaitis GA. Upper extremity assessment in tetraplegia: the importance of differentiating between upper and lower motor neuron paralysis. *Arch Phys Med Rehabil*. 2016;97(6):S97-S104.

3. Oberlin C, Ameur NE, Teboul F, Beaulieu J-Y, Vacher C. Restoration of elbow flexion in brachial plexus injury by transfer of ulnar nerve fascicles to the nerve to the biceps muscle. *Tech hand up extrem surg*. 2002;6(2):86-90.

4. Kahn LC, Moore AM. Donor activation focused rehabilitation approach: maximizing outcomes after nerve transfers. *Hand Clin*. 2016;32(2):263-277.

5. Wangdell J, Bunketorp-Käll L, Koch-Borner S, Fridén J. Early active rehabilitation after grip reconstructive surgery in tetraplegia. *Arch Phys Med Rehabil*. 2016;97(6):S117-S125.

6. Johanson ME, Hentz VR, Smaby N, Murray WM. Activation of brachioradialis muscles transferred to restore lateral pinch in tetraplegia. *J Hand Surgery (American Vomume)*. May-Jun 2006;31(5):747-53.
